# Impact of Upper Extremity Impairment and Trunk Control on Self-Care Independence in Children With Upper Motor Neuron Lesions

**DOI:** 10.1093/ptj/pzab112

**Published:** 2021-04-19

**Authors:** Jeffrey W Keller, Annina Fahr, Jan Lieber, Julia Balzer, Hubertus J A van Hedel

**Affiliations:** Swiss Children’s Rehab, University Children’s Hospital Zurich, Affoltern am Albis, Switzerland; Children’s Research Center, University Children’s Hospital Zurich, Zurich, Switzerland; Doctoral Program Clinical Science, Faculty of Medicine, University of Zurich, Zurich, Switzerland; Swiss Children’s Rehab, University Children’s Hospital Zurich, Affoltern am Albis, Switzerland; Children’s Research Center, University Children’s Hospital Zurich, Zurich, Switzerland; Swiss Children’s Rehab, University Children’s Hospital Zurich, Affoltern am Albis, Switzerland; Children’s Research Center, University Children’s Hospital Zurich, Zurich, Switzerland; Swiss Children’s Rehab, University Children’s Hospital Zurich, Affoltern am Albis, Switzerland; Children’s Research Center, University Children’s Hospital Zurich, Zurich, Switzerland; European University of Applied Sciences (EU | FH)/Erft GmbH, Applied Health Science, Rostock, Germany; Swiss Children’s Rehab, University Children’s Hospital Zurich, Affoltern am Albis, Switzerland; Children’s Research Center, University Children’s Hospital Zurich, Zurich, Switzerland

**Keywords:** Cerebral Palsy, Selective Motor Control, Spasticity, Strength, Trunk Control, Upper Motor Neuron Lesion

## Abstract

**Objective:**

The purpose of this study was to evaluate the relative importance of different approaches to measure upper extremity selective voluntary motor control (SVMC), spasticity, strength, and trunk control for explaining self-care independence in children affected by upper motor neuron lesions.

**Methods:**

Thirty-one patients (mean [SD] age = 12.5 [3.2] years) with mild to moderate arm function impairments participated in this observational study. Self-care independence was evaluated with the Functional Independence Measure for children (WeeFIM). Upper extremity SVMC was quantified with the Selective Control of the Upper Extremity Scale (SCUES), a similarity index (SI_SCUES_) calculated from simultaneously recorded surface electromyography muscle activity patterns, and an accuracy and involuntary movement score derived from an inertial-measurement-unit–based assessgame. The Trunk Control Measurement Scale was applied and upper extremity spasticity (Modified Ashworth Scale) and strength (dynamometry) were assessed. To determine the relative importance of these factors for self-care independence, 3 regression models were created: 1 included only upper extremity SVMC measures, 1 included upper extremity and trunk SVMC measures (overall SVMC model), and 1 included all measures (final self-care model).

**Results:**

In the upper extremity SVMC model (total variance explained 52.5%), the assessgame (30.7%) and SCUES (16.5%) were more important than the SI_SCUES_ (4.5%). In the overall SVMC model (75.0%), trunk SVMC (39.0%) was followed by the assessgame (21.1%), SCUES (11.0%), and SI_SCUES_ (4.5%). In the final self-care model (82.1%), trunk control explained 43.2%, upper extremity SVMC explained 23.1%, spasticity explained 12.3%, and strength explained 2.3%.

**Conclusion:**

Although upper extremity SVMC explains a substantial portion of self-care independence, overall trunk control was even more important. Whether training trunk control and SVMC can translate to improved self-care independence should be the subject of future research.

**Impact:**

This study highlights the importance of trunk control and selective voluntary motor control for self-care independence in children with upper motor neuron lesions.

## Introduction

Achieving independence during self-care activities of daily life is an important goal of neurorehabilitation therapy for children and adolescents affected by upper motor neuron lesions. Such patients can be affected by a multitude of motor signs that impair their ability for independent self-care.^1^ Using the International Classification of Functioning, Disability and Health framework,^2^ associations between measures on the activity level have been studied in children with cerebral palsy (CP), by relating manual ability and functional independence.^3–5^ Furthermore, past studies have explored associations between activity and individual body function measures, such as strength,^6^^,^^7^ spasticity,^8^^,^^9^ selective voluntary motor control (SVMC),^10–12^ and trunk control.^13^^,^^14^ However, studies addressing the relative importance of these body function measures on activities, especially self-care independence, are lacking.

Despite the potential importance of SVMC, tools with the sole purpose of measuring SVMC of the upper extremities have only been established recently.^15^ SVMC has been defined as the ability to activate muscles in a selected pattern to meet demands of a voluntary movement or posture.^1^

Therefore, the Selective Control of the Upper Extremity Scale (SCUES)^16^ and Test of Arm Selective Control^17^ both require the performance of isolated joint movements and penalize involuntary movements occurring, such as mirror or trunk movements and movements of other joints. By recording surface electromyography (sEMG) simultaneously during the performance of the SCUES, we turned it into an objective, neurophysiological assessment, capable of evaluating the similarity of muscle activation patterns (similarity index: SI_SCUES_) between patients and neurologically healthy participants.^18^ Furthermore, we created an objective SVMC assessment game (and propose the term “assessgame”), which uses inertial measurement units. Patients perform a tracking task using isolated joint movements recorded by 2 sensors, while the other sensors capture simultaneously occurring involuntary movements.^19^

The goal of this study was 3-fold. The first goal was to answer the question whether various approaches measuring upper extremity SVMC are of different relative importance in explaining self-care independence in the daily life of children affected by upper motor neuron lesions. Our a priori formulated hypothesis was that the SCUES and assessgame would account for more of the self-care independence variance than the SI_SCUES_, because both measures, by testing for movement, account for strength, which the SI_SCUES_ does not. The second was to investigate the relative importance of upper extremity and trunk SVMC for self-care. We created an overall SVMC model by including the upper extremity SVMC measures and the selective trunk control part of the Trunk Control Measurement Scale (TCMS). We expected trunk SVMC to be relatively more important than upper extremity SVMC. Third, we created a final self-care model to investigate the relative importance of upper extremity SVMC, strength, and spasticity, as well as trunk control (ie, static control, selective control, and dynamic reaching) in explaining self-care independence variance. We expected trunk control to account for most variance because studies have indicated its profound importance.^14^^,^^20^ Thereafter, upper extremity SVMC was hypothesized to account for most variance, followed by strength and finally spasticity.

## Methods

### Participants

Inpatients and outpatients of the Swiss Children’s Rehab in Affoltern am Albis, Switzerland were recruited by convenience sampling. Inclusion criteria were a diagnosed upper motor neuron lesion, age 6 to 18 years, and the ability to understand and follow simple instructions. Patients were excluded if they had undergone any surgical intervention or received botulinum toxin injections in the upper extremities in the past 6 months. Neurologically intact adults aged 18 to 50 years made up the reference group for the assessgame and the SI_SCUES_, because involuntary movements can be physiologically early (<10 years) and later (>50 years) in life.^21^^,^^22^ Neurologically intact adults represent movement mastery without any involuntary movements and therefore the maximal possible score for the sensor-based measures. Furthermore, neurologically intact children between the ages of 6 and 18 years served as a reference group for the strength measurements.

The characteristics age, sex, and handedness (writing hand) were assessed for all participants. Additionally, we measured the weight, height, and arm length of patients and their healthy peers. Furthermore, for patients, we recorded the diagnosis and the more affected or nondominant hand, which was determined by an occupational therapist. Severity of upper extremity involvement was classified with the Manual Ability Classification System (MACS).^23^ Patients who handle objects successfully with ease in daily life receive a MACS score of I, whereas patients with a MACS level V do not handle objects at all. Severity of lower extremity and trunk involvement was classified with the gross motor function classification system (GMFCS).^24^ GMFCS I indicates that although balance, speed, and coordination may be impaired, children are able to perform gross motor skills such as running, jumping, and climbing stairs without holding on to the railing. Children at GMFCS level V have only limited abilities to control their head and trunk postures against gravity and are thus transported in a manual wheelchair in all settings. Children with CP are routinely assessed with the MACS and GMFCS by medical professionals in our rehabilitation center. For this study, patients with other diagnoses were also classified.

Written informed consent was obtained from legal guardians and participants aged 14 years and older. Underage participants gave oral consent. All methods were in accordance with the necessary guidelines and approved by the Ethics Commission of the canton of Zurich, Switzerland (PB_2016_01843).

### Measurements

The total time to perform all measurements varied between 120 and 150 minutes. Therefore, children participated in 2 to 3 appointments, depending on how many breaks they needed. The first and last appointments were separated by no more than 48 hours.

#### Self-Care Independence in Daily Life

The Functional Independence Measure for children (WeeFIM) indicates how independently patients actually perform tasks in daily life and not what the patient might be capable of. The WeeFIM covers 3 domains (self-care, mobility, and cognition). The self-care domain is made up of 6 items, namely eating, grooming, bathing, toileting, and dressing of the upper and lower body. Although bladder and bowel control are part of the self-care domain, we excluded them for this study. The WeeFIM rates independence on a scale from 1 (total assistance needed) to 7 (complete independence, performs task timely and safely). The maximal score for the 6 self-care items was 42. The WeeFIM can be administered to children aged 0.6 to 7 years but can also be used to evaluate children with disabilities above the age of 7 years,^25^ including patients with upper motor neuron lesions.^26^^,^^27^ The primary nurse responsible for the patient observes the patient performing the activities of daily living, and provides assistance if necessary. For the items that the primary nurses cannot observe (eg, during the night shift), they ask the nurses responsible during that shift. The score should reflect an everyday performance; therefore, if the nurses feel that the patient is just having a bad day, they can repeat the measurement the next day. The WeeFIM is assessed weekly in our rehabilitation center by experienced nurses who, after being initially certified, go through a recertification process every 2 years. For outpatients, the nurse in charge of the certification process at our center conducted interviews with the primary care givers, which for this study were the patients’ parents.

#### Measures of SVMC

The SCUES measures SVMC of the shoulder abduction/adduction, lower arm (pronation/supination), and elbow, wrist, and fingers flexion/extension. The SCUES is a body function measure and was validated in children with CP,^16^ but is also valid and reliable in children with upper motor neuron lesions.^28^ The SCUES has a 4-point ordinal scale. A score of 0 indicates that no selective movement is possible, whereas 3 indicates that the range of movement of the target joint exceeded 85% of the passive range, and no additional movements occurred at any other joints or the trunk. The maximal total score of the SCUES is 30.

**Figure 1:**
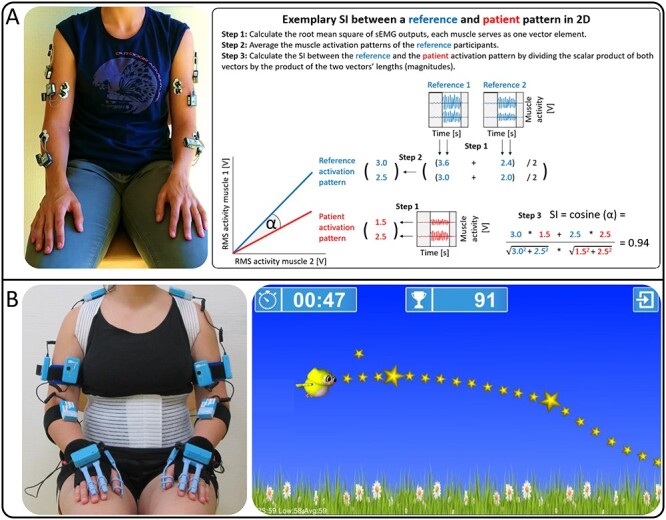
Different approaches quantifying SVMC. A, Sensor placement of the SI_SCUES_ and an exemplary calculation in 2 dimensions (ie, with 2 muscles). For simplicity, we display only 2 reference participants, when in fact there were 33. The RMS of the raw sEMG signal of each muscle results in an activation pattern, where every muscle serves as an element. The gray areas of the sEMG signal are nonmovement phases and thus excluded. The reference activation pattern is made up of multiple individual patterns, which are averaged, illustrated here with “reference 1 and 2.” Finally, the activation patterns are used as vectors and the cosine of the angle between those vectors is the similarity index. The SI_SCUES_ uses 10 sEMG sensors, which are placed on both arms to generate activation patterns. B, Sensor set-up of the assessgame and a screenshot of the game with the predefined trajectory that patients are asked to follow. While patients use isolated target joint movements to steer the avatar on the trajectory, the other sensors capture involuntary movements. Thus, the assessgame results in a metric for the accuracy and involuntary movements. RMS = root mean square; sEMG = surface electromyography; SI = similarity index; SI_SCUES_ = similarity index of the Selective Control of the Upper Extremity Scale; SVMC = selective voluntary motor control.

Prior to performing the SCUES, sEMG electrodes were placed bilaterally (Fig. 1A) on the deltoideus pars acromialis muscle, triceps brachii caput longum muscle, biceps brachii muscle, extensor carpi ulnaris muscle, and flexor digitorum superficialis muscle, in accordance with the Surface ElectroMyoGraphy for the Non-Invasive Assessment of Muscles (SENIAM) guidelines,^29^ where available. This sensor set-up allows the capturing of muscle coactivations ranging from synergistical patterns (ipsilateral muscle activation) to mirror movements (contralateral activations of homologous muscle groups).

For each SCUES movement, we recorded the muscle activations of these 10 muscles, resulting in an activation pattern. Each activation pattern of each SCUES movement was compared with the reference pattern of neurologically intact adults performing the same SCUES movements. This resulted in a similarity index for each SCUES movement (see Fig. 1A for a step-by-step example using 2 muscles for visualization purposes). Finally, we averaged the similarity indices of all 10 SCUES movements to create the SI_SCUES_. The SI_SCUES_ can range from 0, indicating maximal dissimilarity, to 1, reflecting an identical pattern. The SI_SCUES_ is valid and reliable for children with upper motor neuron lesions.^18^

Finally, we created an assessgame (Fig. 1B) in collaboration with Reha-Stim Medtech AG (Schlieren, Switzerland). We published an in-depth description of the game^30^ and established its validity in children with upper motor neuron lesions.^18^ The assessgame uses 10 inertial measurement units and 6 bend sensors that record flexion and extension of fingers and thumbs (see Fig. 1B for sensor positioning). Participants play a tracking task within 90% of their active range of motion. The assessgame tests shoulder abduction/adduction, lower arm (pronation/supination), and elbow, wrist, and fingers flexion/extension. Patients were asked to use isolated joint movements (ie, instructed to steer the avatar only with the target movement/joint) to follow the predefined trajectory, meanwhile the sensors recorded involuntary movements (any other movement occurring in the nontarget joints). Therefore, the assessgame can split SVMC into 2 metrics, one quantifies the accuracy of the target joint performance (ie, the error score), and the other quantifies magnitude and frequency of involuntary movements of the upper extremity joints and the trunk (ie, the involuntary movement score). A score of 0 indicates a perfect performance, ie, the participant followed the target path perfectly and showed no movements in joints other than the ones shown by the neurologically intact adults. The higher the score, the worse the performance on both metrics.

#### Trunk Control

Trunk control was assessed with the TCMS,^31^ a valid^32^ and reliable^33^ tool for children with a range of neuromotor disorders, including upper motor neuron lesions. The TCMS measures body function level on an ordinal scale and is made up of 15 items evaluating static (5 items, 20 points) and dynamic (10 items) sitting balance. Dynamic sitting balance is further divided into dynamic reaching (3 items, 10 points) and selective control (7 items, 28 points). Therefore, the total score can range from 0 to 58, with higher scores indicating a better trunk control.

#### Spasticity

An experienced occupational therapist evaluated the muscle tone of the shoulder abductors/adductors, lower arm pronators/supinators, and the elbow, wrist, and finger flexors/extensors using the Modified Ashworth Scale (MAS). The MAS is a body function measure and rates muscle tone on a 4-point ordinal scale.^34^ A score of 0 indicates no increase in muscle tone, whereas a score of 4 stands for complete rigidity. Therefore, the maximally possible sum score for the bilaterally assessed MAS was 80. The MAS uses an intermediate step score (1+) between a score of 1 and 2. For the analysis, we set this at 1.5 points.

### Strength Measurements and Analysis

We used a hand-held dynamometer (microFET 2; Hoggan Scientific, Salt Lake City, UT, USA) to quantify the body function measure strength of the shoulder abductors, elbow flexors/extensors, lower arm pronators/supinators, wrist extensors, and finger flexors (Hydraulic Hand Dynamometer; Baseline® Evaluation Instruments, Irvington, NY, USA). The exact dynamometer and joint positioning can be found in the Supplementary Appendix (description above Table S1).

For each muscle group (eg, shoulder abductors, elbow extensors), we expressed the patients’ strength values as percentage of the expected strength value obtained from neurologically intact children. Using linear regression analyses and 10-fold cross-validation (ie, 90% of the data to create a model, 10% to validate it), only bodyweight remained as regressor (among sex, height, arm length, and the interaction term of bodyweight and sex) for predicting the expected strength value (Suppl. Table S1). Finally, before averaging the strength percentages over all muscle groups, the elbow flexion/extension as well as the lower arm pronation/supination were averaged into 1 value each.

### Sample Size Calculation

The sample size calculation was based on the study by Likhi et al,^20^ which investigated the association between the Trunk Impairment Scale and the total Functional Independence Measure score (includes self-care, locomotion, sphincter control, and cognition) in adults with stroke. Despite expecting more explained variance, because we included upper extremity body functions, we used the R^2^ = 35.8% they found for the multiple linear regression (“pwr.f2.test” function of the package “pwr” [v 1.3–0]^35^): effect size = R^2^/(1 − R^2^) = 0.358/(1 − 0.358) = 0.55; regressors in model = 5; type I error = 0.05; power = 0.9. Thus, a sample size of 30 participants was calculated to be sufficient.

### Statistical Analysis

The statistical analysis was performed in R (version 3.6.2)^36^ using the packages “relaimpo” (v 2.2–3, non-US version),^37^ and “boot” (v 1.3–23).^38^ Spearman rank correlations were used to understand how regressors were correlated. To answer which upper extremity SVMC measure is most important for explaining self-care independence, we used the relative importance measure proposed by Lindeman, Merenda, and Gold,^39^ henceforth referred to as LMG. The LMG is suited for multiple regression models, where regressors are correlated, because it averages sequential sums of squares for all possible orderings of regressors (eg, for 4 regressors 4! = 4 × 3 × 2 × 1 = 24 orderings). Therefore, the LMG is preferable to other measures of relative importance because it is independent of the regressors’ entry order.^36^ We used the bias-corrected and accelerated bootstrap method^40^ with 2000 bootstrap samples to calculate 95% CIs of the LMG measure and differences between LMG measures of different regressors. The same LMG and bootstrapping methods were used to determine the relative importance of regressors in all following models.

For the overall SVMC model, the TCMS SVMC subscore was added to the first model. In the final self-care model, the most important upper extremity SVMC regressor was carried forward to determine the relative importance among upper extremity SVMC, spasticity, strength, and trunk control (all subscores combined) for self-care independence. Age was entered in all models as covariate. To better understand the relationships among regressors and the dependent variable, we additionally calculated how much variance a regressor uniquely explains (entering the regressor last) and how much variance is shared (total variance explained minus all unique contributions).

### Role of the Funding Source

The funders played no role in the design, conduct, or reporting of this study.

## Results

Of the 38 patients that gave informed consent, 7 had to be excluded—6 due to compliance issues (age and cognition), and 1 patient was too severely impaired to perform the assessments. The 31 patients (11 females) had a median age of 12.5 years (interquartile range [IQR]: 9.2-15.1 years) varying between 7.5 and 17.4 years. They were diagnosed with cerebral palsy (n = 19; bilateral spastic = 13, unilateral spastic = 4, ataxic = 2), stroke (n = 8), traumatic brain injury (n = 2), encephalitis (n = 1), and partial temporal lobe resection (n = 1). The MACS levels were I (n = 9), II (n = 11), III (n = 10), and IV (n = 1). The GMFCS levels were as follows: GMFCS I: n = 11; II: n = 4; III: n = 10; IV: n = 6. Results of the various measures are presented in the Table. The strength measurements reference group was made up of 32 neurologically intact children (16 females) with a median age of 11.0 years (IQR: 8.6-13.8 years). The reference group for the similarity index consisted of 33 neurologically intact adults (18 females), with a median age of 32.5 years (IQR: 27.9-38.3 years).

**Table:** Patient Body Function and Activity Measures^a^

Measures	Median	1st to 3rd Quartile	Range
Self-care: WeeFIM SC	33.0	20.5–39.0	12–42
SVMC measures:			
SCUES	21.0	16.5–26.5	11–30
SI_SCUES_	0.88	0.81–0.93	0.66–.98
AG accuracy	2.1	1.4–2.6	0.9–3.8
AG involuntary movements	3.1	1.5–4.6	0.7–11.7
Trunk control: TCMS	44.0	27.0–51.0	0–58
Spasticity: MAS	2.0	0.0–3.8	0–13
Strength: dynamometry, %	76.4	53.3–85.0	19.6–127.5

^*a*^AG = assessgame; MAS = Modified Ashworth Scale; SCUES = Selective Control of the Upper Extremity Scale; SI_SCUES_ = Similarity Index of the SCUES; SVMC = selective voluntary motor control; TCMS = Trunk Control Measurement Scale; WeeFIM SC = pediatric Functional Independence Measure self-care domain (without bladder and bowel control).

**Figure 2:**
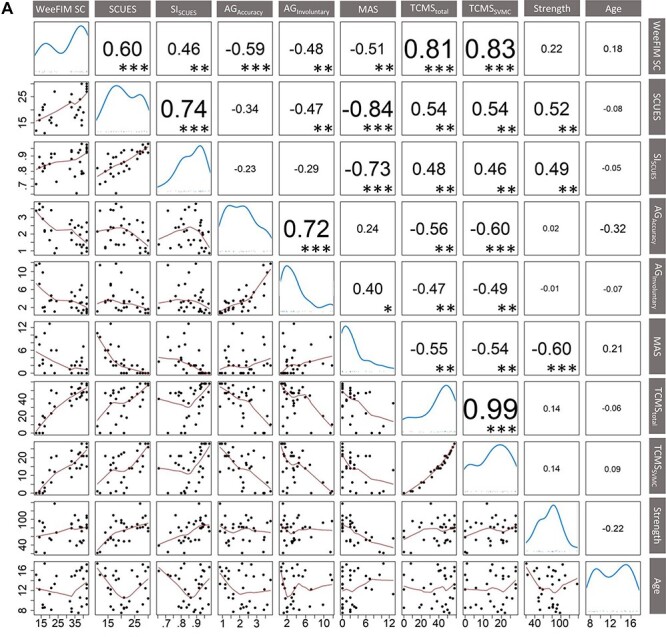
Correlation matrix and corresponding Spearman rank correlation coefficients of all regression variables. The diagonal of the correlation matrix displays density distribution (analogous to histograms) of all measures used in the different models. The dots in the scatter plot represent the mean value of each patient. The red lines in the scatter plots are locally weighted scatterplot smoothing (LOWESS) curves with default settings of the function pairs in R (graphics package v 3.6.2). Absolute correlation coefficients higher than 0.36, 0.46, and 0.58 have associated *P*-values (indicated by asterisks) that are lower than 0.05 (^*^), 0.01 (^**^), and 0.001 (^***^), respectively. AG = assessgame; MAS = Modified Ashworth Scale; SCUES = Selective Control of the Upper Extremity Scale; SI_SCUES_ = Similarity Index of the SCUES; SVMC = selective voluntary motor control; Strength = strength value in % of neurologically intact peers; TCMS = Trunk Control Measurement Scale; TCMS_SVMC_ = selective voluntary motor control subscore of the TCMS; WeeFIM SC = pediatric Functional Independence Measure self-care domain (without bladder and bowel control).

The Spearman rank correlation coefficients displayed in Figure 2 indicate how regressors and self-care independence are correlated. Some regressors were highly correlated (eg, SCUES and MAS) whereas others were not correlated at all (eg, assessgame accuracy and strength).

**Figure 3:**
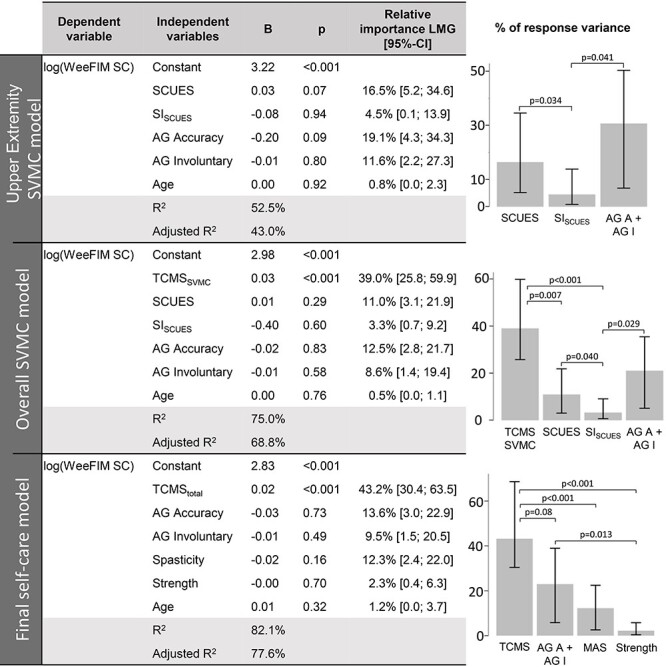
Multiple linear regression models for explaining self-care independence in daily life. Model regressors and their relative importance for explaining the log-transformed WeeFIM self-care domain (without bladder and bowel control) with bias-corrected and accelerated bootstrap 95% CIs. The *P*-values for regressor comparison (right column) are displayed only if they are below 0.1. Because age was entered as a covariate, it was not compared with other regressors. The upper extremity SVMC model indicates that the assessgame and SCUES are more important than the SI_SCUES_. The overall SVMC model reveals that the TCMS_SVMC_ regressor on average explains significantly more variance than the SCUES and SI_SCUES_. Furthermore, the SCUES and assessgame are still more important than SI_SCUES_. In the final self-care model, trunk control is the most important regressor, followed by upper extremity SVMC and spasticity, and, finally, strength. 95%–CI = bias-corrected and accelerated bootstrap 95% confidence interval; AG = assessgame; AG A = assessgame accuracy; AG IM = assessgame involuntary movements; B = regressor coefficient estimate; LMG = relative importance measure proposed by Lindeman, Merenda, and Gold^39^; log() = logarithmic transformation; MAS = Modified Ashworth Scale (spasticity); p = *P* value; R^2^ = variance of the log-ransformed WeeFIM self-care domain explained by the independent variable; SCUES = Selective Control of the Upper Extremity Scale; SI_SCUES_ = Similarity Index of the SCUES; Strength = strength value in % of neurologically intact peers; SVMC = selective voluntary motor control; TCMS_total_ = Trunk Control Measurement Scale total score; TCMS_SVMC_ = selective voluntary motor control subscore of the TCMS; WeeFIM SC = pediatric Functional Independence Measure self-care domain (without bladder and bowel control).

**Figure 4:**
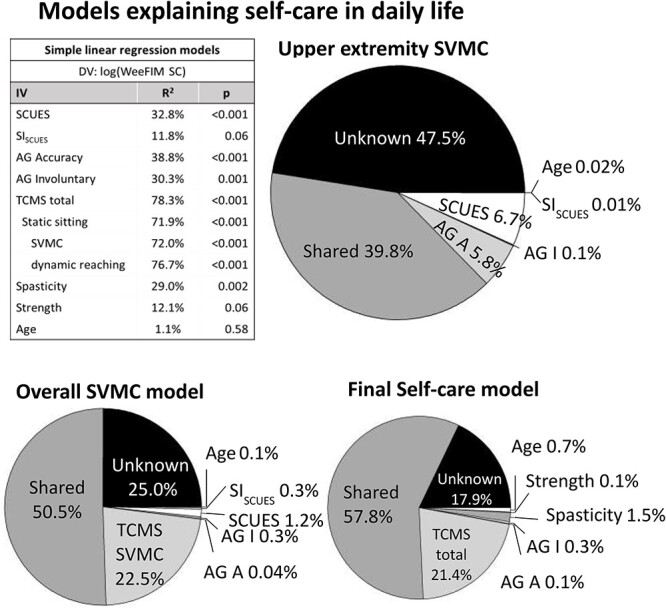
Unique and shared contributions of regressors explaining the variance of self-care in daily life. The table displays the individual contributions of all regressors, if they are entered into the model individually (simple regression), to explain the WeeFIM self-care domain (without bladder and bowel control). For example, the SCUES explains 32.8% of the variance of the log-transformed WeeFIM self-care domain. The pie charts indicate which proportion of the log-transformed WeeFIM self-care variance is unaccounted for (unknown), explained (100% minus unknown proportion, or Fig. 3), shared (total variance explained minus all unique contributions), and contributed uniquely (variance explained, when entered last) by the regressors. For example, the “upper extremity SVMC model” indicates that although the SCUES alone explains 32.8% of the variance, in this model the SCUES only contributes 6.7% uniquely and shares the rest (32.8% – 6.7% = 26.1%) with the other regressors. AG = assessgame; AG A = assessgame accuracy; AG I = assessgame involuntary movements; DV = dependent variable; IV = independent variable; LMG = relative importance measure proposed by Lindeman, Merenda, and Gold^39^; log() = logarithmic transformation; SCUES = Selective Control of the Upper Extremity Scale; R^2^ = variance of the log-transformed WeeFIM self-care domain explained by the IV; SI_SCUES_ = Similarity Index of the SCUES; Strength = strength value in % of neurologically intact peers; SVMC = selective voluntary motor control; TCMS = Trunk Control Measurement Scale; TCMS_SVMC_ = selective voluntary motor control subscore of the TCMS; WeeFIM SC = pediatric Functional Independence Measure self-care domain (without bladder and bowel control).

The regression models explaining the variance of the WeeFIM self-care domain can be found in Figure 3. The upper extremity SVMC model explains approximately 50% of the variance of the WeeFIM self-care domain and indicates that the assessgame is slightly more important than the SCUES but not by a significant margin. Moreover, both the SCUES (16.5%) and assessgame (30.7%) explain significantly more variance than the SI_SCUES_ (4.5%). The overall SVMC model, which explains 75% of self-care independence variance, reveals that the TCMS SVMC subscore (39.0%) is significantly more important than the SCUES (11.0%) and SI_SCUES_ (3.3%). The SCUES and assessgame (21.1%) are still significantly more important than the SI_SCUES_. For the final self-care model, the assessgame was carried forward as upper extremity SVMC measure; however, the SCUES gives almost identical results (Suppl. Fig. S1). The final self-care model explains about 80% of the variance of the WeeFIM self-care. Trunk control (43.2%) is the most important regressor followed by the upper extremity SVMC (23.1%) and spasticity (12.3%) measures, and, finally, the strength (2.3%) measure. Trunk control is significantly more important than spasticity and strength. The difference between the upper extremity SVMC and trunk control shows a trend towards significance. Upper extremity SVMC is significantly more important than strength. Depending on the regressors entered, there is an overlap or shared variance between the regressors of 39.8% and 57.8% (Fig. 4). In the upper extremity SVMC model, regressors share much of the variance explained, yet the assessgame accuracy score and the SCUES contribute about 6% to 7% uniquely. Trunk SVMC and overall trunk control make the highest unique contributions in the overall SVMC and final self-care model, respectively.

## Discussion

This study evaluated the impact of reduced SVMC in particular, as well as impairments in trunk control and upper extremity spasticity and strength on independence in self-care activities of daily life in children with upper motor neuron lesions. All measures of upper extremity SVMC combined explained about half of the variance in the WeeFIM self-care domain, with the assessgame and SCUES explaining most. Adding TCMS SVMC to the upper extremity SVMC model resulted in the overall SVMC model, which explained 75%, with TCMS SVMC being the most important regressor. When evaluating other upper extremity impairments and trunk control, the final self-care model explained approximately 80% of the variance of self-care independence. Trunk control explained most of the variance, followed by upper extremity SVMC, spasticity, and lastly strength.

Regarding the variance of self-care independence explained by upper extremity SVMC, it becomes clear that the SCUES/SI_SCUES_ and the assessgame metrics share a lot of variance because they all assess a similar construct and are therefore correlated. Yet, although there is overlap between the various SVMC assessments, the assessgame and the SCUES do seem to cover slightly different aspects of SVMC relevant for self-care independence. This is indicated by the unique variance the 2 assessments still explain after accounting for the overlap, even with the SI_SCUES_ included in the model (Fig. 4 “Upper extremity SVMC model”). One reason is that the assessgame might resemble situations of everyday life closer than the SCUES. When explaining the assessgame, we explicitly asked participants to move only the target joint to follow the predefined trajectory. When involuntary movements occurred, the measurement was not repeated, unlike the SCUES, where participants are allowed to repeat the task while trying to suppress involuntary movements.^16^

The observation that the SI_SCUES_ accounted for less of the variance of daily self-care independence than the assessgame or the SCUES might be explained by the SI_SCUES_ capturing the underlying muscle activations independent of movement.^41^ Such an assessment is integral for understanding which motor strategies are used for achieving a specific movement. However, the fact that a movement can be performed at all, even with compensational muscle activities, might have more bearing for independence in daily life. For example, patients that are relatively weak might exhibit physiological muscle activation patterns but not be independent during self-care activities.

The overall SVMC model further highlights the importance of SVMC for self-care independence, especially trunk SVMC. However, as can be appreciated by the simple regressions in Figure 4 and in Supplementary Figure S1, all subcomponents of the TCMS are of similar importance and highly correlated (Spearman correlations between 0.88 and 0.90). Therefore, we discuss the importance of overall trunk control in the context of the final self-care model below.

Concerning the final self-care model, this is, to our knowledge, the first study evaluating the importance of such a set of regressors in children with upper motor neuron lesions. Our results indicate that by far the most important regressor for explaining self-care independence was trunk control. Past studies either highlighted the importance of trunk control for independence in adults with stroke^42–44^ or the importance for upper extremity function on independence in children with CP^3–5^ and adults with stroke.^45^ The most comparable study to ours was done by Likhi et al,^20^ who concluded that correlations were highest between the total Functional Independence Measure score (includes self-care, locomotion, sphincter control, and cognition) and trunk impairment, followed by upper extremity impairments in patients with stroke. An explanation for why trunk control could account for large parts of variability of self-care independence in daily life may be that trunk control provides the stable base that is needed for the upper extremity to function correctly in the first place.^46^ A statement that might have merit also for the lower extremity.^47^ Highlighting this, Santamaria et al^14^ conducted a study where they supported children with CP with varying levels of trunk dysfunction at different levels of the trunk. They concluded that the motor performance of children affected by trunk dysfunction benefitted from external trunk support. Systematic reviews indicate that direct and indirect trunk training can improve certain aspects of trunk control for patients with CP^48^ and stroke.^49^ However, although a specific trunk rotation device^50^ was found to provide greater improvements in terms of trunk control compared with conventional neurorehabilitation in patients with stroke, the total Functional Independence Measure score improved equally in both groups.^51^ Therefore, the question whether different methods of trunk control training can translate to improved self-care independence remains unanswered and should be the subject of future evaluations.

Past studies have reported the importance of manual ability, that is, the MACS, on self-care.^3–5^ However, the comparison of individual components of upper extremity function has not been reported so far. Our results imply that SVMC and spasticity are more important than strength. An explanation for this finding could be that, despite patients varying widely with respect to their average strength values, very few could not move their joints at all. In fact, of 310 tested joint movements, only 4 movements of 2 patients received a SCUES score of 0, indicating no visible joint movement. Inversely, because patients had enough strength to perform upper extremity movements, more strength was less important than SVMC and spasticity for self-care independence. Chiu et al^52^ made the same observation when accounting for upper extremity activity variance with elbow strength, spasticity, contracture, and coordination in children with CP. However, patients with stroke exhibiting lower levels of strength might be more impaired by this weakness than loss of dexterity.^53^

### Limitations

The fact that patients in this study were mildly to moderately impaired, as indicated by the MACS (only 1 patient had level 4), also limits its generalizability to patients with more severe impairments. The cognitive requirements to participate in this study excluded most patients with severe motor deficits. It would, however, be interesting to evaluate the impact of trunk control, SVMC, strength, and spasticity, using simpler (eg, manual muscle testing instead of dynamometry) and more time-efficient approaches, to include also patients that are more severely affected. Future studies might want to consider such an approach.

Moreover, we used neurologically intact children as reference only for the strength metric and not for the others. This prevents us to a certain extent from disambiguating the effects of neurological impairment and age-related development in the patient group. Whereas the WeeFIM, SI_SCUES_, MAS, and strength measure (corrected for in the used metric) do not (no longer) show a relationship with age, the SCUES and both assessgame metrics do. For the TCMS, we do not have data on this topic, but the score might improve even in neurologically intact children of the age range we included. We tried to statistically account for this by adding age as a covariate to all models. However, such a correction can never remedy the issue entirely.

Finally, certain movements that are relevant for self-care independence, for example, shoulder flexion/extension or specific thumb movements, were not assessed. Future studies evaluating the relative importance of individual joints/movements/muscle groups on self-care independence could, according to these results, focus on fewer tests and include more joints.

### Conclusion

Among the upper extremity SVMC measures (total variance explained: 52.5%), the SCUES and assessgame were most important for explaining self-care independence of children affected by upper motor neuron lesions, accounting for slightly different portions of variance. Adding trunk SVMC increased the explained variance of self-care independence to 75%. In the final self-care model (82.1%), trunk control accounted for the largest proportion of variance of self-care independence, followed by upper extremity SVMC, spasticity, and strength. Trunk control was significantly more important than spasticity and strength. Upper extremity SVMC was significantly more important than strength in patients with mild to moderate impairments. The question whether different methods of trunk control and upper extremity SVMC training can translate to improved self-care independence should be the subject of future evaluations.

## Supplementary Material

Appendix_FINAL_FILE_jk_pzab112
