# Supplementary material for: Impact of Upper Extremity Impairment and Trunk Control on Self-Care Independence in Children With Upper Motor Neuron Lesions
Source: Phys Ther. 2021 Apr 19;101(8):pzab112. doi: 10.1093/ptj/pzab112 (PMC8407597; doi:10.1093/ptj/pzab112)
Supplement: Appendix_FINAL_FILE_jk_pzab112 [file appendix_final_file_jk_pzab112.docx]

# Appendix

During shoulder abduction measurements, the elbow was flexed at 90 degrees, while the dynamometer was positioned on the upper arm slightly proximal of the lateral epicondyle. For the elbow flexion, the lower arm was supinated (if possible) and the dynamometer placed proximally of the ulnar styloid process. The lower arm was placed on the table with cushioning. For the elbow extension, the arm remained on the table with the patient pressing down on the dynamometer, which was placed proximally of the ulnar styloid process with the lower arm in a neutral position (resembling a chopping motion). The wrist extension measurements were done with the participant’s hand clenched to a fist and the dynamometer placed proximally of the knuckles while the lower arm rested on a 10 cm foam block. Finger flexion strength was evaluated with the elbow in a 90-degree angle, while the participants tried to clench their fist. Measurement were repeated three times, starting with the dominant/less affected side and alternating sides after each measurement. We used the strongest of the three measurements for each side.

| **Table S1:** Linear regression models to predict expected strength values in Newton per joint. | | | | | | | | | | |
| --- | --- | --- | --- | --- | --- | --- | --- | --- | --- | --- |
|  | |  |  | Possible predictors | | | | |  |  |
| Models | | TF | Interc. | Sex [0;1] | Weight [kg] | Weight*Sex | Height [cm] | Arml. [cm] | Adj. R^2^ | Influential cases |
| D. SH abd | | log | 3.350 |  | 0.029 |  |  |  | 0.84 | ID 31 |
| Nd. SH abd | | log | 3.538 |  | 0.027 |  |  |  | 0.85 | ID 31 |
| D. ELB flex | | no | -32.262 |  | 3.831 |  |  |  | 0.90 | ID 25, ID 31 |
| Nd. ELB flex | | no | -33.192 |  | 3.811 |  |  |  | 0.86 | ID 31 |
| D. ELB ext | | no | -8.519 |  | 2.729 |  |  |  | 0.76 | no effect on model |
| Nd. ELB ext | | no | 2.735 |  | 2.342 |  |  |  | 0.75 | ID 17 |
| D. LA supi. | | log | 1.648 |  | 0.028 |  |  |  | 0.81 | no effect on model |
| Nd. LA supi | | log | 1.726 |  | 0.026 |  |  |  | 0.83 | no effect on model |
| D. LA pro | | log | 2.275 |  | 0.020 |  |  |  | 0.64 | no effect on model |
| Nd. LA pro | | log | 2.292 |  | 0.018 |  |  |  | 0.62 | no effect on model |
| D. WR ext | | no | -8.089 |  | 2.250 |  |  |  | 0.87 | ID 6, ID 25 |
| Nd. WR ext | | no | -7.218 |  | 2.126 |  |  |  | 0.87 | ID6, ID 25 |
| D. FI flex | | log | 4.496 |  | 0.024 |  |  |  | 0.86 | ID25, ID31 |
| Nd. FI flex | | log | 4.442 |  | 0.024 |  |  |  | 0.84 | no effect on model |
| *Abbreviations: TF = applied transformation; Interc. = intercept; Sex = female (1), male (0); * = interaction; Arml. = armlength; Adj. = adjusted; log = logarithmic; D. = less affected/dominant; Nd. = more affected/non-dominant; SH = shoulder; ELB = elbow; LA = lower arm; WR = wrist; FI = finger; abd = abduction; flex = flexion; ext = extension; supi = supination; pro = pronation.* | | | | | | | | | | |
|  |  |  |  |  |  |  |  |  |  |  |

**Since age was accounted for in the main models directly, age and its interactions were not included in the above strength models.**


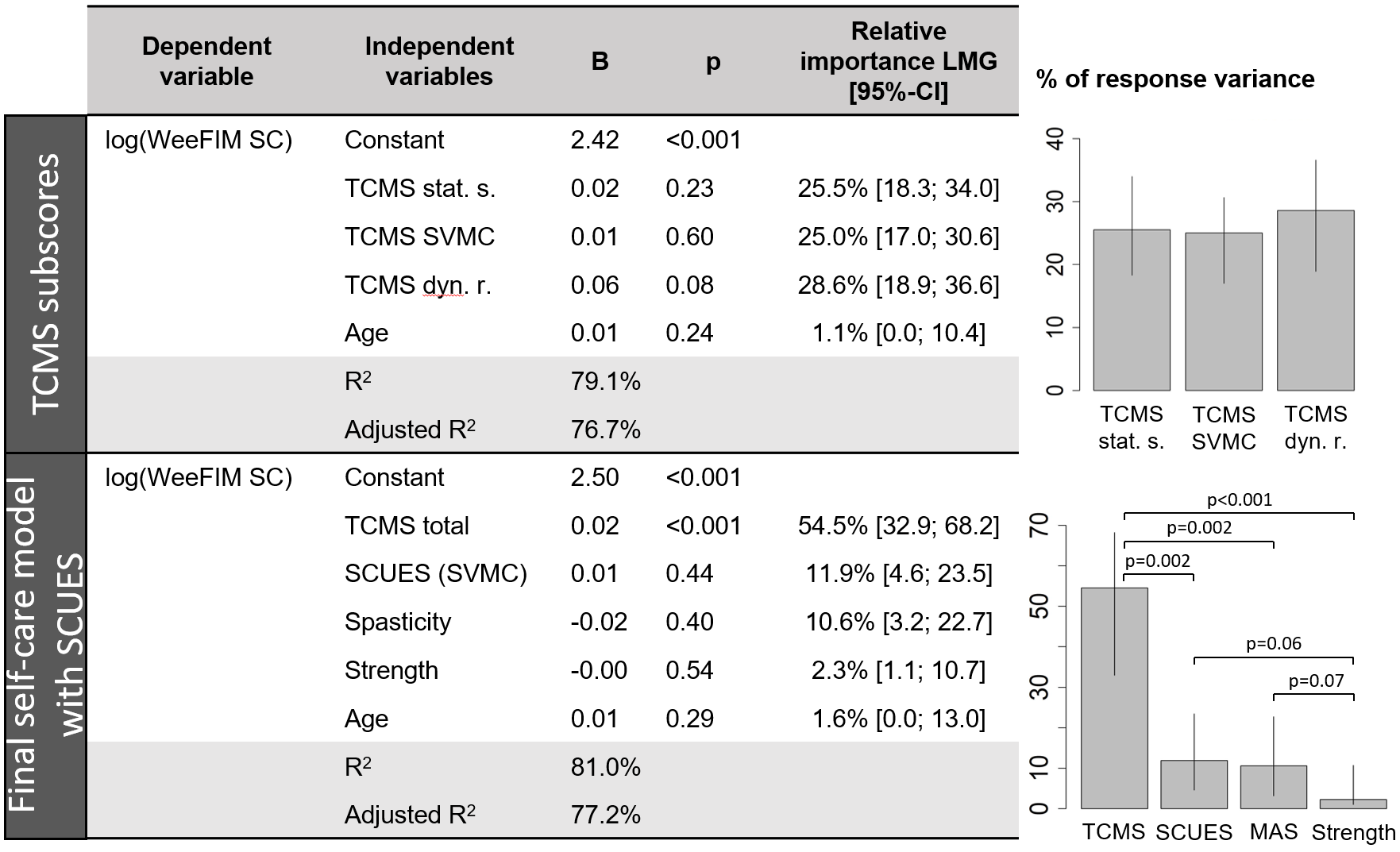


**Figure S1: Multiple linear regression models for explaining self-care independence in daily life. Model regressors and their relative importance for explaining the log-transformed WeeFIM self-care domain (without bladder and bowel control) with bias-corrected and accelerated bootstrap 95%-confidence intervals. The p-values for regressor comparison (in bar plots) are displayed only if they are below 0.1. Since age was entered as a covariate, it was not compared to other regressors.**

***Abbreviations: B = regressor coefficient estimate; p = p-value; LMG = relative importance measure proposed by Lindeman, Merenda, and Gold*** ^37^***; 95%-CI = bias-corrected and accelerated bootstrap 95% confidence interval; log() = logarithmic transformation; WeeFIM SC= pediatric Functional Independence Measure self-care domain (without bladder and bowel control); SVMC = selective voluntary motor control; SCUES = Selective Control of the Upper Extremity Scale; MAS = Modified Ashworth Scale (spasticity); TCMS stat. s. = Trunk Control Measurement Scale static sitting subscore; TCMS SVMC = selective voluntary motor control subscore of the TCMS; TCMS dyn. r. = dynamic reaching subscore of the TCMS; Strength = strength value in % of neurologically intact peers.***
